# Purkinje Cell Compartmentation in the Cerebellum of the Lysosomal Acid Phosphatase 2 Mutant Mouse (*Nax* - Naked-Ataxia Mutant Mouse)

**DOI:** 10.1371/journal.pone.0094327

**Published:** 2014-04-10

**Authors:** Karen Bailey, Maryam Rahimi Balaei, Ashraf Mannan, Marc R. Del Bigio, Hassan Marzban

**Affiliations:** 1 Department of Human Anatomy and Cell Science, Manitoba Institute of Child Health (MICH), Faculty of Medicine, University of Manitoba, Winnipeg, Manitoba, Canada; 2 Institute of Human Genetics, University Medical Center Goettingen, Georg-August University, Goettingen, Germany; 3 Department of Pathology, Faculty of Medicine, University of Manitoba, Winnipeg, Manitoba, Canada; Tokyo Medical and Dental University, Japan

## Abstract

The *Acp2* gene encodes the beta subunit of lysosomal acid phosphatase, which is an isoenzyme that hydrolyzes orthophosphoric monoesters. In mice, a spontaneous mutation in *Acp2* results in severe cerebellar defects. These include a reduced size, abnormal lobulation, and an apparent anterior cerebellar disorder with an absent or hypoplastic vermis. Based on differential gene expression in the cerebellum, the mouse cerebellar cortex can normally be compartmentalized anteroposteriorly into four transverse zones and mediolaterally into parasagittal stripes. In this study, immunohistochemistry was performed using various Purkinje cell compartmentation markers to examine their expression patterns in the *Acp2* mutant. Despite the abnormal lobulation and anterior cerebellar defects, zebrin II and PLCβ4 showed similar expression patterns in the *nax* mutant and wild type cerebellum. However, fewer stripes were found in the anterior zone of the *nax* mutant, which could be due to a lack of Purkinje cells or altered expression of the stripe markers. HSP25 expression was uniform in the central zone of the *nax* mutant cerebellum at around postnatal day (P) 18–19, suggesting that HSP25 immunonegative Purkinje cells are absent or delayed in stripe pattern expression compared to the wild type. HSP25 expression became heterogeneous around P22–23, with twice the number of parasagittal stripes in the *nax* mutant compared to the wild type. Aside from reduced size and cortical disorganization, both the posterior zone and nodular zone in the *nax* mutant appeared less abnormal than the rest of the cerebellum. From these results, it is evident that the anterior zone of the *nax* mutant cerebellum is the most severely affected, and this extends beyond the primary fissure into the rostral central zone/vermis. This suggests that ACP2 has critical roles in the development of the anterior cerebellum and it may regulate anterior and central zone compartmentation.

## Introduction

Lysosomal acid phosphatase (LAP) is one of many acid hydrolases responsible for the degradative function of lysosomes [1]. This isoenzyme consists of an alpha and beta subunit. The beta subunit is encoded by the *Acp2* gene. The precursor of Acp2 is a membrane bound protein that is converted to soluble mature Acp2 after it is delivered to the lysosome [2], where it hydrolyzes orthophosphoric monoesters into alcohol and phosphate [3]. Apart from degradation, Acp2 is also critical for normal lysosomal functioning through its removal of recognition signals from other lysosomal proteins [4]. The importance of proper lysosomal function is highlighted by the numerous human diseases that occur due to mutations in lysosomal enzymes which can result in maldevelopment (dysmorphia), underdevelopment (hypoplasia) and/or progressive degeneration of cells (e.g. [5]–[7]). In particular, *ACP2* has been associated with progressive supranuclear palsy [8] and ACP2 activity levels are known to be increased in juvenile neuronal ceroid lipofuscinosis, a disease caused by mutations in the lysosomal protein CLN3 (Batten disease: [9]). Mice with a targeted disruption of *Acp2* (interrupted in exon 7, with truncation of the open reading frame encoding 243 of 423 amino acids of wild-type LAP) have a mild phenotype with generalized lysosomal storage in the central nervous system and kidney [10]. A spontaneous autosomal recessive mutation in *Acp2* (replacement of guanine with adenine at position 740 in exon 7, resulting in conversion of a glycine into glutamic acid at position 244 of the protein) is responsible for cerebellar and skin malformations in *naked-ataxia* (*nax*) mice [11]. Surprisingly, although neither of these mutants have detectable Acp2 enzymatic activity, only *nax* has obvious developmental abnormalities of the brain, suggesting a novel function for the modified Acp2 protein.

Cerebellar morphology is based upon a characteristic array of lobes, lobules and folia, with a central region called the vermis flanked on either side by hemispheres (e.g. [12]). Based on differential gene expression, the cerebellum is compartmentalized anteroposteriorly into four transverse zones: the anterior zone (AZ: ∼ lobules I–V), the central zone (CZ: ∼lobules VI–VII: with two sub-zones – see [13]), the posterior zone (PZ: ∼lobules VIII + dorsal IX) and the nodular zone (NZ: ∼ventral lobule IX + lobule X) (e.g., [14]). Each zone is further divided mediolaterally into parasagittal stripes [14], [15]. Although many cerebellar cell types are aligned in a zone and stripe pattern, it has been suggested that much of the cerebellar patterning is built around a Purkinje cell (Pc) scaffold [16]. Examination of Pc compartmentation marker expression patterns has been used to explore cerebellar defects of several mutant mouse models including *dreher* [17], *weaver* [18], *reeler* [19], *cerebellar deficient folia* [20], and *lurcher* [21].

*Acp2* is ubiquitously expressed in mouse tissue, with high levels expressed in the brain and testes [22]. In the brain, *Acp2* expression predominates in cerebral pyramidal neurons, choroid plexus epithelial cells and cerebellar Pcs [22]. We have shown that *Acp2* expression in cerebella of C57B/6 mice is dynamically regulated throughout development [23]. Perinatally, *Acp2* expression is localized in the caudal mesencephalon and diffusely expressed in the developing cerebellum. By postnatal day (P) 10, expression is localized to all Pcs and by the second postnatal week only a subset of Pcs expresses *Acp2* in a zone and stripe pattern typical of cerebellar topography.

In this study, immunohistochemistry was performed using several Pc markers to examine morphological alterations and gene expression patterns in the *nax* mutant cerebellum. The results suggest that there are changes in the morphogenetic program in the *nax* mutant mouse and altered Pc organization that may contribute to the dysmorphic and hypotrophic/hypoplastic cerebellum.

## Materials and Methods

### Animal maintenance and tissue processing

Animal procedures conformed to institutional regulations and the *Guide to the Care and Use of Experimental Animal*s from the Canadian Council for Animal Care. This study was approved by the University of Manitoba Animal Care Committee (ACC). All experiments conformed to international guidelines on the ethical use of animals. All efforts were made to minimize the number of animals used and their suffering.

*Nax* mutant embryos were obtained from the Institute of Human Genetics in the University Medical Center, Georg-August University, Goettingen, Germany. A colony was established in the Genetic Model Center at the University of Manitoba by breeding mice (C57BL/6) heterozygous for the *nax* mutation (homozygote/heterozygote/wild type ratio was ∼25%:50%: 25%, respectively). Animal husbandry followed the regulations of The Canadian Council on Animal Care (CCAC). The animals were kept at room temperature and relative humidity (18–20°C, 50–60%) on a 12∶12 light and dark cycle. Animals were grouped in standard polycarbonate cages with dust-reduced wood shavings as bedding and food and water were provided ad libitum.

Phenotypically, *nax* mutant mice were easily distinguished from littermate controls based on the delayed appearance or lack of hair over the entire body, smaller stature and ataxia. To confirm genotypes, PCR (polymerase chain reaction; DNA extracted from mouse tails) was performed according to Mannan et al (2004) using the following primers: Acp4F (GCACTCTGTGCCTTCTCCAT) and Acp4R (CTGGGAGATTTGGGCAACTA).

Adult and postnatal developing mice from P6 to P26 (wt; n = 18 and *nax*; n = 19) were deeply anesthetized with isoflurane (30% isoflurane in propylene glycol) anaesthesia in a desiccator jar inside a fume hood. Adequate depth of anesthesia was confirmed by observing that there was no change in respiratory rate associated with manipulation and toe pinch or corneal reflex. Upon confirmation that a suitable anesthetic plane (no response to stimulation) was attained, a thoracotomy was performed in supine position. Trans-cardiac perfusion was then carried out using normal saline followed by 4% paraformaldehyde (PFA) in 0.1 M phosphate buffer (pH 7.4). The brains were removed and post-fixed in 4% PFA at 4°C for a minimum of 24 hours. The fixed tissue was washed in phosphate buffered saline (PBS) and cryoprotected by immersion in 10% (2 hours), 20% (2 hours) and 30% (overnight) buffered sucrose solutions. Tissue was embedded in optimal cutting temperature (OCT) medium for 30 min at room temperature and then stored at −80°C until use. Serial transverse and sagittal sections were cut at 30 µm and stored free-floating in PBS at 4°C.

### Antibodies

Two anti-calbindin (CaBP) antibodies were used;

- Rabbit polyclonal anti-calbindin D-28K antiserum (anti-CaBP, diluted 1∶1000, Swant Inc. Bellinzona, Switzerland) produced against recombinant rat calbindin D-28K. In the cerebellum, CaBP is exclusively expressed in Pcs (e.g., [24], [25]).
- Mouse monoclonal anti-calbindin (anti-CaBP, diluted 1∶1000, Swant Inc. Bellinzona, Switzerland) raised against chicken calbindin D-28K. Immunohistochemistry yielded Pc specific staining identical to that previously reported; e.g., [26].

Zebrin II

- Anti-zebrin II (ZII) (a gift from Dr. Richard Hawkes, University of Calgary, Calgary, Alberta, Canada) is a mouse monoclonal antibody produced by immunization with a crude cerebellar homogenate from the weakly electric fish *Apteronotus*. It was used directly from spent hybridoma culture medium (diluted 1∶200) [15], [27], [28].

Phospholipase Cβ4

- Anti-phospholipase Cβ4 (PLCβ4) is a rabbit polyclonal antibody that recognizes the PLCβ4 protein, which is expressed in a subset of Pcs (Abcam Inc.: ab103279; diluted 1∶100 and also a gift from Dr. Richard Hawkes, University of Calgary, Calgary, Alberta, Canada, and Dr. Masahiko Watanabe; Department of Anatomy, Hokkaido University School of Medicine, Sapporo 060-8638, Japan diluted 1∶500) (for details see [29], [30]).

Heat shock protein 25

- Anti-small heat shock protein 25 (HSP25) is a rabbit polyclonal antibody obtained from StressGen, Victoria BC, Canada (diluted 1∶1000). Hsp25 is expressed in a subset of Pcs (for details see [31]).

Lysosomal acid phosphatase 2

- Anti-ACP2 (4B5; diluted 1∶1000) is a mouse monoclonal antibody raised against a full recombinant ACP2 protein of human origin that recognizes an epitope on lysosomal acid phosphatase 2 (Acp2) (Santa Cruz Biotechnology, Inc.; sc100344).

Neuron specific nuclear protein

- Anti-neuron specific nuclear protein (NeuN) is a rabbit polyclonal antibody that recognizes the N terminus of NeuN (Millipore: Catalog No. ABN78; diluted 1∶1000). In this study it was used to identify postmitotic granule cells (Weyer and Schilling, 2003).

Parvalbumin

- Anti- parvalbumin (PV) is a rabbit polyclonal antibody obtained from Swant Inc. Bellinzona, Switzerland (Code No. PV25; diluted 1∶1000). It was used to identify stellate and basket cells in the molecular layer [32]–[34].

Caspase-3

- Anti- cleaved caspase-3 is a rabbit monoclonal antibody that recognizes the endogenous levels of the large fragment (17/19 kDa) of activated caspase-3 resulting from cleavage adjacent to Asp175 (Cell Signaling Technology: Apoptosis Antibody Sampler Kit (Mouse Preferred) #9930; cleaved caspase-3 (Asp175) (5A1E) Rabbit mAb #9664; diluted 1∶200). In this study it was used as an indicator of apoptosis (e.g. [35]).

### Section Immunohistochemistry

Peroxidase and double-label fluorescent immunohistochemistry were carried out on free-floating cerebellar sections as described previously [13]. Briefly, for peroxidase immunohistochemistry, tissue sections were washed with PBS and endogenous peroxidase activity was blocked using 0.3% H_2_O_2_ in PBS for 20 min, followed by washing in PBS. An antigen retrieval technique was used for anti-PLCB4 where sections were dehydrated and then rehydrated through a series of graded methanol prior to the blocking of peroxidase activity. Sections were then blocked for 1 hr in blocking solution (10% normal goat serums and 0.05% Triton x-100 in 0.1 M PBS) and incubated in primary antibody (diluted in blocking solution) overnight at room temperature. After washing with PBS, sections were incubated for 1 hr at room temperature in horse radish peroxidase (HRP)-conjugated goat anti-rabbit or goat anti-mouse immunoglobulin (Millipore: Catalog No. AP307P and AP308P; diluted 1∶500 in blocking solution). As an isotype negative control, sections were processed as above but addition of primary antibody was replaced by a suitable IgG isotype. Tissue was washed with PBS again and peroxidase activity was revealed using diaminobenzidine (DAB) as the chromogen. Sections were dehydrated through a series of alcohols, cleared with xylene and coverslipped using Krystalon mounting medium (Harleco). For anti-HSP25 and anti-ACP2 immunohistochemistry, the Vectastain ABC Staining Kit was used to enhance antibody detection. Sections were incubated in biotinylated secondary antibody for 1 hr, washed with PBS, incubated in ABC reagent for 1 hr and washed again in PBS before development using DAB.

For double-immunofluorescence a similar method as above was used, however, tissue was co-incubated in primary antibodies overnight at room temperature and then incubated in Alexa Fluor 488 goat anti-mouse IgG and Alexa Fluor 594 goat anti-rabbit IgG (Life Technologies: Catalog No. A-11029 and A-11012; diluted 1∶1000 in blocking solution) for 1 hr at room temperature. Selected sections were counterstained with DAPI for 15 min at room temperature. Sections were then washed in PBS and coverslipped with Fluorsave (Calbiochem: Catalog No. 345789).

### Whole mount Immunohistochemistry

Whole mount immunohistochemistry was performed on the cerebellum according to Sillitoe and Hawkes (2002) [36] except that PBS containing 0.2% skim milk (Nestlé Foods Inc., North York ON, Canada) plus 0.1% Triton-X 100 (Sigma, St. Louis MO, USA) was used as the blocking solution (PBSMT). In addition, 5% dimethyl sulfoxide (DMSO) was added to PBSMT for the overnight blocking step and after staining was revealed the tissue was washed in PBS and stored in PFA.

A heat induced epitope retrieval method was used to re-expose the PLCB4 epitopes. Entire cerebella were placed in boiling 10 mM sodium citrate buffer (95°C; pH 6.0) for 60 min and then allowed to cool in the buffer at room temperature. Specimens were rinsed in PBS prior to beginning the whole mount immunohistochemistry process.

### Quantitative data

To obtain brain weight measurements, brains fixed in 4% PFA at P19 were cut apart from the spinal cord at the level of the caudal medulla oblongata and weighed on a laboratory scale (n = 5).

The rostrocaudal length of the unfolded vermis in the *wt* and *nax* cerebellum was measured along the pial surface at three postnatal ages (P6; n = 2, P12; n = 3 and P17; n = 3). For each cerebellum 2 midline sections were measured using Zen software.

To quantify the Pcl/ml thickness, sagittal sections of the vermis immunostained with CaBP were used. In the *nax* mutant, measurements were taken from the junction of the pcl/ml and putative granular layer to the pial surface. In the wild type, measurements were taken from the junction of the Pcl and granular layer to the pial surface.

In order to quantify Pcs, we counted the number of Pcs in 7–8 fields in each sagittal section immunostained with CaBP (P6; n = 2, P12; n = 3, and P17; n = 3; 2 sections per cerebellar vermis). We estimated the "linear density"  =  number of PCs/mm.
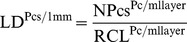



LD^Pcs/1 mm^  =  Linear Density of Pcs per 1 mm

NPcs^Pc/ml layer^  =  Number of Pcs in sagittal section of the vermis

RCL^Pc/ml layer^  =  Rostrocaudal length of the sagittal section of the vermis

Data are presented as mean ± Standard error of mean (SEM) by using student t-test and two way ANOVA followed by Bonferroni post-hoc. Student's t-test was used to compare two groups. Two-way ANOVA was applied for two independent variables, was assessed on a dependent variable, and followed by Bonferroni post-hoc. *P*<0.05 was considered statistically significant.

### Figure Preparation

For bright field microscopy an Olympus BH-2 microscope was used and images were captured using Image-Pro Expression software. For fluorescence microscopy a Ziess Lumar.V12 stereomicroscope was used to capture images of entire cerebellar sections using AxioVision 4 software. For high magnification fluorescence microscopy a Ziess Z1.Imager with AxioVision 4 software and a Zeiss LSM 700 confocal microscope with Zen software were used to obtain images. Images were cropped, corrected for brightness and contrast, and assembled into montages using Adobe Photoshop CS5 Version 12.

## Results

### Phenotype of the *nax* mutant

**Figure 1:**
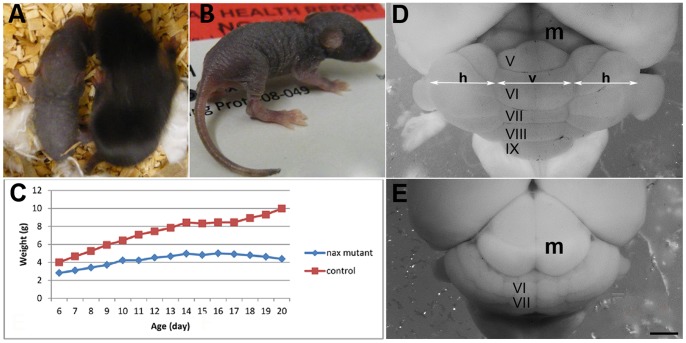
Physical description of the *nax* mutant mouse. **A–B**) *nax* mutant and wild type sibling at P10. The *nax* mutant is characterized by lack of hair over the entire body and a smaller stature compared to the wild type sibling. **C**) Growth curve of *nax* mutant mice in comparison with wild type siblings showing severe growth retardation during postnatal development. **D–E**) Dorsal view of wild type sibling (**D**) and *nax* mutant (**E**) cerebella at P19. (**D**) The wild type cerebellum developed normally with a lobulated vermis (v; middle arrow) in the middle and hemispheres (h; arrow in each side) on each side. (**E**) An underdeveloped vermis is prominent in the small cerebellum of the *nax* mutant and the boundary between the vermis and each hemisphere is not obvious. The midbrain (m) can clearly be seen from the dorsal aspect due to the small cerebellum. Individual lobules in the vermis are indicated in Roman numerals. Scale bar: E = 1 mm (D, E).

**Figure 2:**
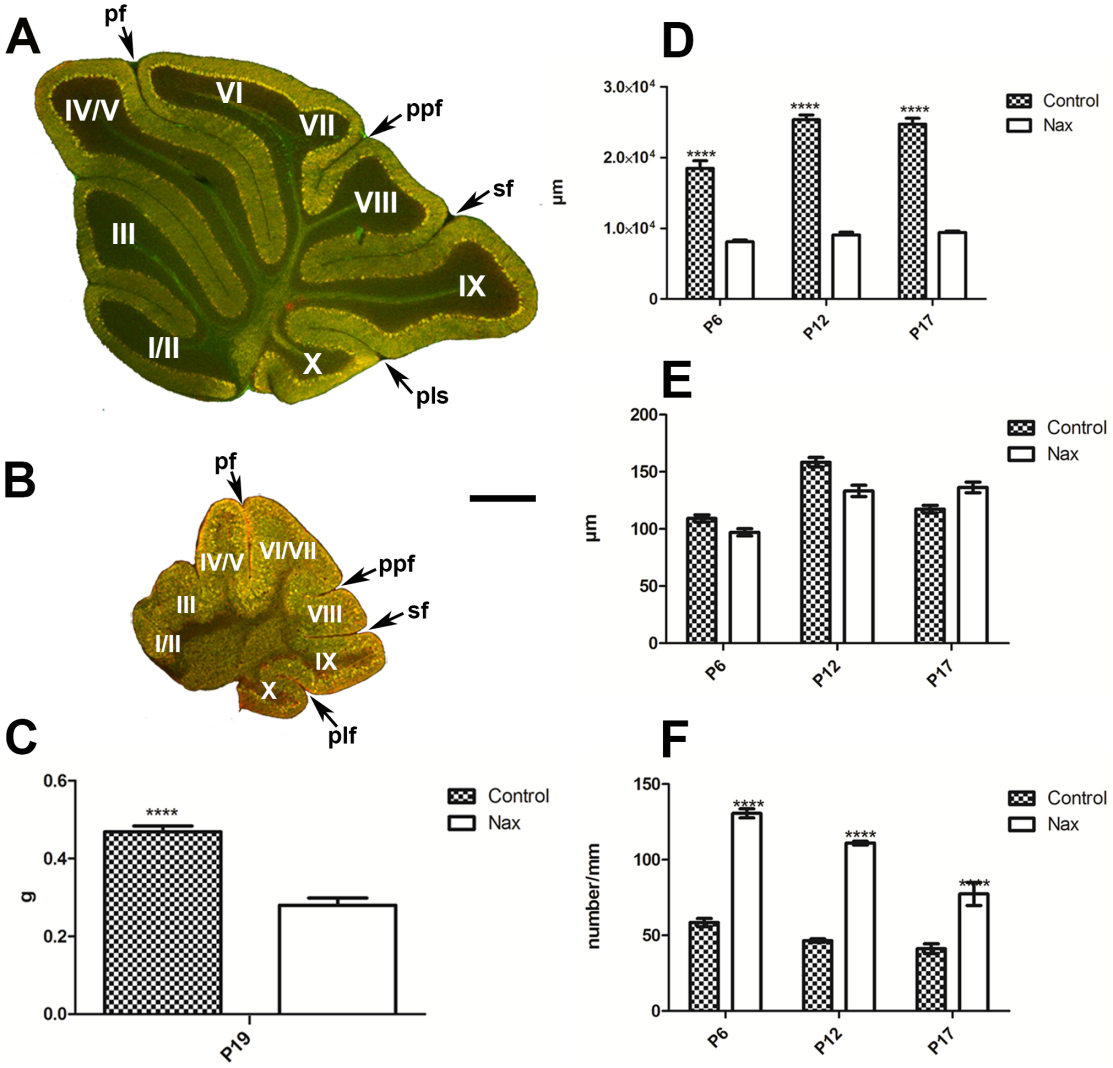
Sagittal sections through the *nax* mutant and wild type cerebella at P17 immunostained with CaBP and statistical graphs. The lobules are indicated by Roman numerals. **A**) The cerebellum in the wild type developed normally with a lobulated vermis. **B**) The cerebellum in the *nax* mutant is smaller and underdeveloped, however the principal lobules in the vermis are present. **C**) Average brain weights (in grams (g) mean ± SEM) in the nax mutant and wild type at P19 indicate a significant decrease in weight of the nax brain. **D**) The rostrocaudal length of the nax mutant and wild type cerebellum at P6, P12, and P17, shows that the nax cerebellum is much smaller than the wild type. **E**) The Pcl/ml thickness in the nax mutant is smaller than that of the wild-type at P6 and P12, however it becomes larger at P17. **F**) The linear density of Pcs per 1 mm significantly decreases from P6 to P17 in the nax mutant, however it is higher than the linear density of Pcs in wild-type at every age. Scale bars: 500 um.

Mice homozygous for the *nax* mutation had several phenotypic abnormalities as previously described in detail [11]; the most obvious were small stature, whole body alopecia (Fig. 1A–B) and ataxic movement. The average life span of *nax* mice was about P20, with the oldest living to P26. *Nax* mice weighed on average around 50% less than wild type siblings and a decrease in weight usually occurred around P18, with death shortly following (Fig.H1C). Macroscopic examination of the cranial cavity revealed a smaller brain in the *nax* mutant (∼0.28 g) compared to wild type siblings (∼0.47 g) (Whole fixed brains weighed at P19, n = 5 (Fig. 2C; P<0.0001). The *nax* cerebellum appeared to be underdeveloped with a potentially hypoplastic or absent vermis compared to wild type siblings (Fig. 1D, E).

### Morphology of the *nax* cerebellum

A midsagittal section through the vermis of the wild type cerebellum at P17 showed the basic pattern of ten lobules (Fig. 2A). A similar view of the *nax* cerebellum revealed the presence of the main lobules and fissures (Fig. 2B), but the rostrocaudal length of the cerebellum was severely shortened compared to the wild type (Fig. 2D; *P*<0.0001). Although all the main lobules could be identified in the *nax* mutant, development of the lobules appeared to be reduced or retarded (Fig. 2B). In addition, the white matter tracts in the *nax* cerebellum were narrowed or absent in the core of the lobules.

In the wild type, Pcl/ml thickness in the vermis increased from P6 to P12 and then decreased by P17, whereas in the *nax* mutant it had a slight increase from P6 to P17 (Fig. 2E). The decrease in the wt could be explained by the disappearance of the external granule layer around the end of the third postnatal week. This effect would not be expected to be as obvious in the *nax* cerebellar cortex as the external granule layer is much smaller or absent (data not shown). Thickness of the Pcl/ml in the *nax* mutant was smaller than the wild type at P6 and P12, but larger at P17 (Fig 2. E). Calculation of the linear density of Pcs showed that the number of Pcs per mm of cortex was significantly decreased from P6 to P17 in the *nax* cerebellum (Fig. 2F; *P*<0.0001), however the density in the *nax* mutant was higher than the wild type at all ages. This could be explained by the decreased cerebellar size in the *nax* mutant resulting in more Pcs per unit area. The decrease in Pc density as age increased in the *nax* mutant suggests that Pcs are dying.

**Figure 3:**
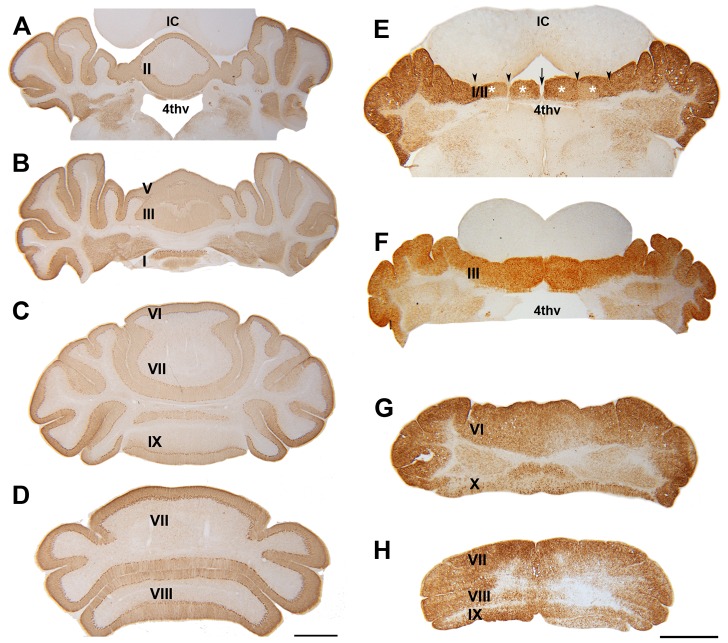
Frontal sections of P21 wild type (A–D) and *nax* (E–H) cerebella immunostained with CaBP. **A–D**) The wild type cerebellum shows normal lobules and the central vermis is prominent with hemispheres on either side. **E–H**) From the rostral section (E) to the caudal section (H), the *nax* cerebellum shows obvious signs of a hypoplastic vermis compared to the wild type. In lobules I/II (E), the hemispheres fail to fuse completely in the midline, but fusion becomes more obvious as the sections progress posteriorly. The underdeveloped anterior lobules of the *nax* cerebellum have shallow rostrocaudal fissures (arrow head) causing a blocked appearance of the cortex (white asterisk) that is symmetrical about the midline (arrow). A putative vermis area may be present in caudal sections of the *nax* cerebellum, but all that is noticeable is that the medial part (putative vermis) is smaller in relation to the hemispheric part, compared to the wild type. Individual lobules in the vermis are indicated in Roman numerals. Abbreviation: 4thv =  fourth ventricle; IC =  Inferior colliculus. Scale bar: D = 1 mm (A–D); H = 1 mm (E–H).

Histological analysis of transverse sections immunostained for CaBP at P21 showed that despite the small size of the *nax* cerebellum, the cerebellar cortex, white matter and cerebellar nuclei were all located normally, with no ectopic Purkinje cells in the white matter (Fig. 3). Examination of transverse sections also revealed signs of a hypoplastic or absent vermis in the *nax* cerebellum. The wild type had a well-developed and prominent vermis (Fig. 3A–B), whereas, the putative vermis of the *nax* cerebellum was severely underdeveloped anteriorly and formed only a narrow connection between the two hemispheres (Fig. 3E–F). In the wild type, as sections progressed caudally, the vermis became larger than the hemispheres in the sagittal plane (Fig. 3C–D). In contrast, in the *nax* mutant a uniform width of the cerebellum was seen along with continuation of the two hemispheres without obvious interruption within the vermis (Fig. 3G–H).

**Figure 4:**
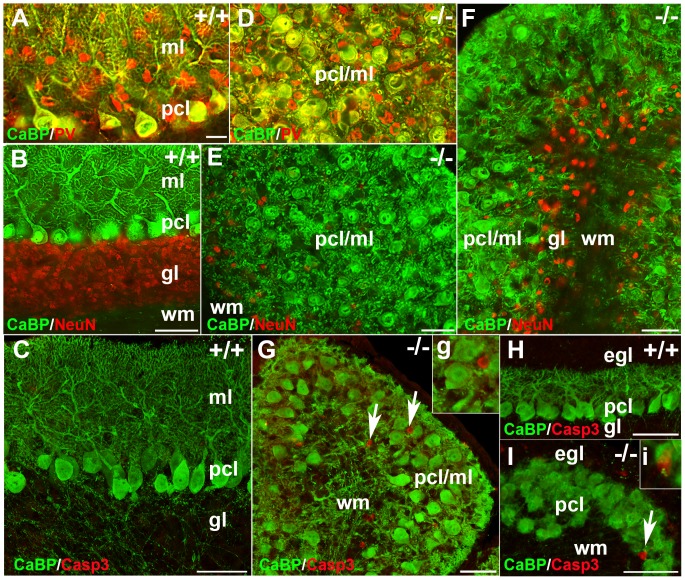
Transverse sections at P21 and sagittal sections at P6 and P17 of the *nax* mutant and wild type cerebellum. **A**) Double immunostaining with CaBP (green) and PV (red) in a transverse section through the cerebellum of the wild type sibling showing inhibitory interneurons and the dendrites of Pcs in the molecular layer (ml) and Purkinje cell soma in the Purkinje cell layer (pcl). **B**) A transverse section through the cerebellum of the wild type sibling double immunostained with CaBP (green) and NeuN (red), showing the three layers of the cortex: molecular layer (ml), Purkinje cell layer (pcl) and granular layer (gl). **C**) Double immunostaining with CaBP (green) and cleaved caspase 3 (red) in a sagittal section of the wild type sibling cerebellum at P17 showing lack of caspase + cells in cerebellum. **D**) Double immunostaining with CaBP (green) and PV (red) in a transverse section through the cerebellum of the *nax* mutant showing that the Pcs fail to form a uniform monolayer. Inhibitory interneurons and Pcs soma are intermingled in the ml; labeled as pcl/ml. **E–F**) Transverse sections through the cerebellum of the *nax* mutant, anteromedially (E; putative anterior vermis) and posterolaterally (F; hemisphere). In the anterior lobe, an apparent lack of the granular layer places the pcl/ml directly in contact with white matter (D). In the posterolateral cerebellum, a small amount of cerebellar granule cells form a granular layer between the pcl/ml and wm (E). Purkinje cells are arranged in different directions. **G**) Double immunostaining with CaBP (green) and cleaved caspase 3 (red) in a sagittal section through the cerebellum of the *nax* mutant at P17 showing that the caspase 3 immunopositive cells (arrow; higher magnification in “g”) are not co-labeled with CaBP+ (Pcs) cells. **H–I**) Double immunostaining with CaBP (green) and cleaved caspase 3 (red) in sagittal sections of wild type cerebellum (H) and *nax* mutant cerebellum (I) at P6 showing that the caspase 3 immunopositive cells (arrow; higher magnification in “I”) are not co-labeled with CaBP+ (Pcs) cells. Scale bar: A = 25 µm (A, D); B, E, F = 50 µm, C, H, J = 50 µm, G = 40 µm.

Immunohistochemistry on P21 transverse sections using anti- CaBP, PV and NeuN revealed severe disorganization of neurons within the cerebellar cortex of the *nax* mutant (Fig. 4). In the wild type, the cerebellar cortex was arranged in three layers: the molecular layer containing Pc dendrites, the Pc layer containing a monolayer of Pc somata, and the granular cell layer (Fig. 4A,B). In the *nax* mutant, the three cortical layers were indistinct (Fig. 4D, E, F). One of the most obvious disruptions was that the Pcs failed to align into a monolayer as seen in the wild type (Fig. 4B, D, E). The orientation of Pcs was also abnormal, with dendrites projecting in several directions rather than exclusively toward the pial surface (Fig. 4B, D, E), making it difficult to determine the presence of a clear molecular layer. Double staining with CaBP and PV showed the existence of stellate and basket cells amongst Pcs in the *nax* cerebellum, indicating that the molecular layer was present with Pcs ectopically located in it (Fig. 4D. An underdeveloped granular layer appeared to be present in the posterior lobe of the *nax* cerebellum, but it was almost absent in the anterior lobe, causing the Pc multi-layer to be in direct contact with the white matter (Fig 4E).

In order to determine if the decrease in Pc density in the *nax* mutant over time was a result of Pc death, cleaved caspase-3 (active) and CaBP double immunostaining was performed at P6, P12 and P17. Cleaved caspase-3 positive apoptotic cells were scattered in the *nax* cerebellar cortex, but all of these cells were small and labeling was not co-localized with Pcs (Fig. 4C, G–I).

**Figure 5:**
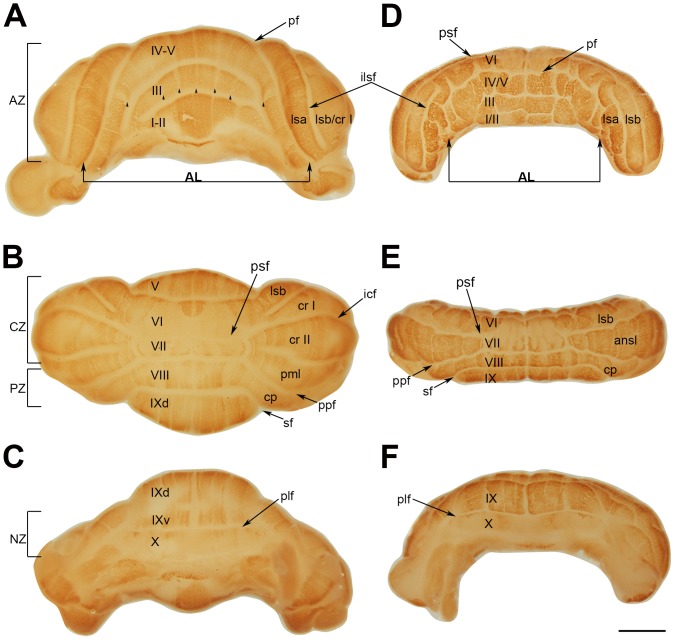
Whole mount immunostaining of wild type (A–C) and *nax* (E–F) cerebella at P8 with PLCβ4. Individual lobules in the vermis are indicated in Roman numerals. **A–C**) The central vermis and cerebellar hemispheres are normally lobulated in the wild type cerebellum. A) The anterior lobe (AL; lobules I–V) is separated from the rest of the cerebellum by the primary fissure (pf). Wide PLCβ4 immunopositive stripes separated by narrow immunonegative stripes are clearly seen in the anterior vermis (indicated by arrowheads). B) The dorsal aspect of the cerebellum shows the posterior lobe (lobules VI–IX). The lobulus simplex (lsa and lsb) is separated from the ansiform lobules (cr I and cr II; crus I and crus II) by the posterior superior fissure (psf). Lobule VIII is separated from lobule VII by the prepyramidal fissure (ppf) and its lateral extension as the copula pyramidis (cp) is separated from lobule IX by the secondary fissure (sf). The vermis of lobule VI–VII is immunonegative with some extension of stripes from the rostral and caudal regions indicating this as a transitional zone. In lobule VIII and IXd, PLCβ4 is expressed in stripes. C) The ventral aspect shows the posterolateral fissure (plf) between lobule IX and X. PLCβ4 is expressed in stripes in lobule IXv and lobule X is immunonegative. **D–F**) D) The small, underdeveloped *nax* cerebellum shows severe anomalies in the anterior lobe that also extend across the primary fissure into lobule simplex “a” (lsa). PLCβ4 is expressed uniformly in the underdeveloped anterior lobe with no obvious stripes. E) From the dorsal aspect, the expression pattern of PLCβ4 shows the presence of a vermis. The vermis of lobule VI and VII is PLCβ4 immunonegative, whereas, their lateral extensions as the lobule simplex b and undivided ansiform lobule appear to have a striped expression pattern. Expression of PLCβ4 is in stripes in lobule VIII and dorsal lobule IX. Lobule X is PLCβ4 immunonegative. Abbreviations: ilsf  = intralobule simplex fissure, icf = intercrural fissure. Scale bar: 1 mm.

Examination of the lobulation in the *nax* cerebellum revealed that the most severe lobular disorganization occurred in the anterior lobe, both in the medial portion (the putative vermis) and the lateral hemispheres. In comparison to the wild type sibling, which had well developed lobules separated by mediolateral fissures (Fig. 5A–C), the *nax* mutant had short, multidirectional fissures present in the anterior cerebellum creating a blocked appearance that was not obvious in the posterior cerebellum (Fig. 5D–F). This blocked appearance was symmetrical about the midline and appeared to be due to folding of the surface of the anterior lobules, which normally appear smooth. In the wild type, rostral and caudal projections of the vermis in lobule V and IX, respectively, were prominent (Fig. 5B). From the anterior aspect, the well-developed anterior lobe vermis of the wild type concealed the vermis of lobule VI, while the hemispheric components of lobule VI (lsa and lsb) protruded on each side (Fig. 5A). Reduced development of the anterior lobe in the *nax* mutant caused the vermis of lobule VI to be visible (Fig. 5D). In the posterior cerebellum of the wild type, lobules were developed and separated by mediolateral fissures; the lobulus simplex was separated from the ansiform lobules (crus I and II) by the posterior superior fissure and the ansiform lobule was separated into crus I and II by the intercrural fissure. Although fissures were directed mediolateraly in the posterior cerebellum of the *nax* mutant, they separated underdeveloped lobules; lobules VI and VII continued laterally as a lobule simplex and an undivided ansiform lobule, lobule VIII continued to the copula pyramidis, and lobule IX was unusually elongated (Fig. 5E–F). The intercrural (or intra-ansiform) fissure was absent in the *nax* cerebellum resulting in a fused crus I and II lobule (Fig. 5E). Overall, these results revealed the presence of maldeveloped lobulation in the *nax* mutant cerebellum.

### Zone and stripe pattern in the *nax* mutant cerebellum

To determine whether patterned zone and stripe expression was altered in association with the lobule and cortical anomalies of the *nax* cerebellum, we examined several cerebellar Purkinje cell stripe markers: ZII, PLCβ4, ACP2 and HSP25. As mentioned, the mouse cerebellum is comprised of four transverse zones and the expression pattern of most molecules studied thus far is striped in the AZ and PZ and uniform in the CZ and NZ [15]. The striped expression patterns of ZII and PLCβ4 are complementary in the mouse cerebellum [29]. ZII immunopositive bands from medial to lateral are termed P1+ to P7+ and they are separated by PLCβ4 immunopositive stripes, named P1- to P6- (e.g. [29], [30]).

Whole mount immunohistochemistry using PLCβ4 was performed at various postnatal ages (P6–P19) and revealed the presence of four transverse zones in the *nax* mutant similar to the wild type sibling; e.g. expression of PLCβ4 at P8 (Fig. 5). PLCβ4 was expressed uniformly in the underdeveloped anterior lobe with no obvious stripes (Fig. 5D), however, wide PLCβ4 immunopositive stripes separated by narrow immunonegative stripes were clearly seen in the cerebellum of the wild type (Fig. 5A, arrowhead). From the dorsal aspect, the expression pattern of PLCβ4 in the nax cerebellum was typical of that of the vermis. The vermis of lobules VI and VII was PLCβ4 immunonegative, whereas, their lateral extensions as the lobule simplex “b” and undivided ansiform lobule appeared to have a striped expression pattern (Fig. 5E). The appearance of lobule simplex “a”, however, resembled the AZ phenotype rather than that of the CZ (Fig. 5D). Similar to the wild type sibling, expression of PLCβ4 was in stripes in the vermis and hemispheric components of lobule VIII (Fig. 5F) and the elongated lobule IX (Fig. 5E, F). Lobule X, the main component of the NZ, was PLCβ4 immunonegative (Fig. 5C, F).

**Figure 6:**
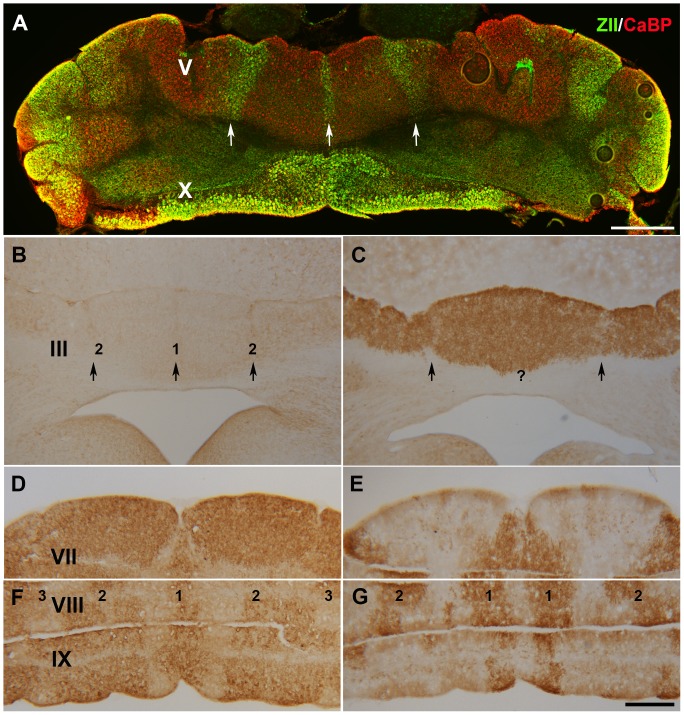
Transverse sections through the *nax* cerebellum immunostained for CaBP, ZII, and PLCâ4. **A**) A transverse section through the *nax* cerebellum double-immunofluorescence stained for CaBP (red) and ZII (green). This shows that ZII is expressed in a subset of Pcs, producing a symmetric pattern of parasagittal stripes (white arrow). **B–G**) Higher-magnification view of ZII and PLCB4 immunoreactivity in a series of transverse sections through the cerebellar vermis of the *nax* mutant. **B–C**) Sections through lobule III immunostained with ZII (B) and PLCβ4 (C). ZII peroxidase deposits produce weak or absent stripes at the midline (1) and on either side (2) (black arrow). PLCB4 expression appears uniform with weak or absent PLCB4- stripes that correspond to the ZII+ stripes. **D–E**) Sections through lobule VII show that Pcs uniformly express ZII (D), while PLCβ4 expression is weak or absent (E). **F–G**) Sections through lobules VIII and IX immunostained with ZII (F) and PLCβ4 (G) reveal that peroxidase deposits are arranged in an array of broad parasagittal stripes with the P1+ (1) stripe in the midline and the P2+ and P3+ stripes on each side of this (2 and 3). The PLCβ4 stripes complementary to the ZII stripes are indicated by 1 (P1-) and 2 (P2-) in G. Scale bars: A = 500 µm; G = 1 mm (B–G).

Using whole mount immunohistochemistry ZII expression was difficult to discern in the anterior nax cerebellum, potentially due to fissures from the hyperfolded lobules, therefore, section immunohistochemistry was performed to examine expression patterns. Double immunostaining for ZII and CaBP confirmed that ZII is expressed in a subset of Pcs and that the alternate pattern of stripes in the *nax* cerebellum was not due to absent Pcs (Fig. 6A). Immunoperoxidase staining for ZII and PLCB4 showed complementary expression in serial sections of the *nax* cerebellum (Fig. 6B–G). ZII+ stripes (Fig. 6B) and PLCB4- stripes (Fig. 6C) were weak or absent in the rostral portion of the anterior lobe. In the caudal portion of the anterior lobe, ZII was clearly expressed in a subset of Pcs, producing a symmetric pattern of parasagittal stripes (Fig. 6A, white arrow). Serial sections through the posterior lobe showed complementary expression patterns of ZII (Fig. 6D, F) and PLCβ4 (Fig. 6E, G).

**Figure 7:**
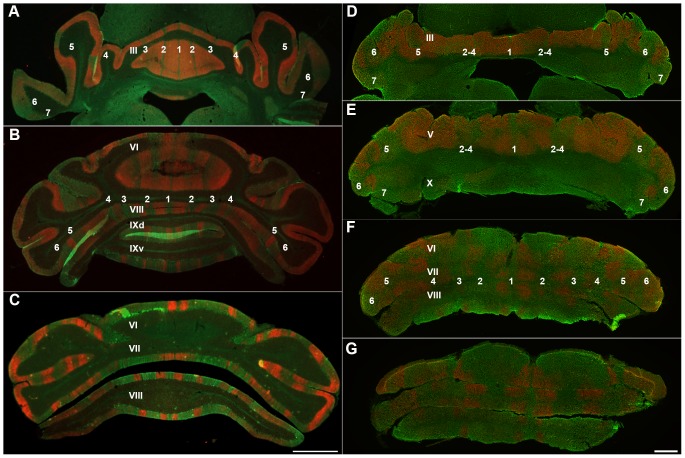
Number of stripes is compared in the wild type (A–C) and *nax* cerebellum (D–G) at P24. Stripe markers are PLCβ4 (red) and ZII (green). **A–C**) Transverse sections through the anterior and posterior lobes of the wild type cerebellum show five clear ZII+ stripes in the vermis that alternate with PLCβ4+ stripes. The stripes in the hemispheres are not as clear as in the vermis, but they are distinguishable and numbered accordingly. **D–G**) In the *nax* mutant cerebellum, fewer stripes of typical gene expression were found. D) ZII+ stripes in the anterior lobe vermis are weak or absent, but are stronger in the hemispheres. E) The pattern of stripes is clearly present in the caudal anterior lobe of the nax mutant, but it appears that either stripes 2–4 are fused or a number of stripes are missing. F, G) The number of stripes in the caudal part of the posterior lobe appears to be similar to the normal pattern. The lobules are indicated by Roman numerals. The ZII+ Purkinje cell stripes are labeled (P1+ to P7+ as 1–7 for clarity). Scale bar: C = 1 mm (A–C); D, E, F, G = 1 mm.

Double immunostaining with PLCβ4 and ZII through the anterior lobe of the *nax* mutant cerebellum revealed an array of alternating ZII/PLCβ4 parasagittal stripes (Fig. 7D–E) similar to the wild type (Fig. 7A–B). However, the number of stripes identified in the AZ of the *nax* mutant was fewer compared to the wild type. It seems that stripes P2+ to P4+ are fused or the subset of Pcs corresponding to some of these stripes either has not formed or it has died. The pattern of PLCβ4/ZII expression in the posterior lobe of the *nax* mutant was similar to the wild type cerebellum (Fig. 7B–C, F–G). The PLCβ4 immunonegative zones (lobules VI/VII (CZ) and X (NZ)) uniformly expressed ZII, while PLCβ4 and ZII were expressed alternately in lobule VIII (PZ).

**Figure 8:**
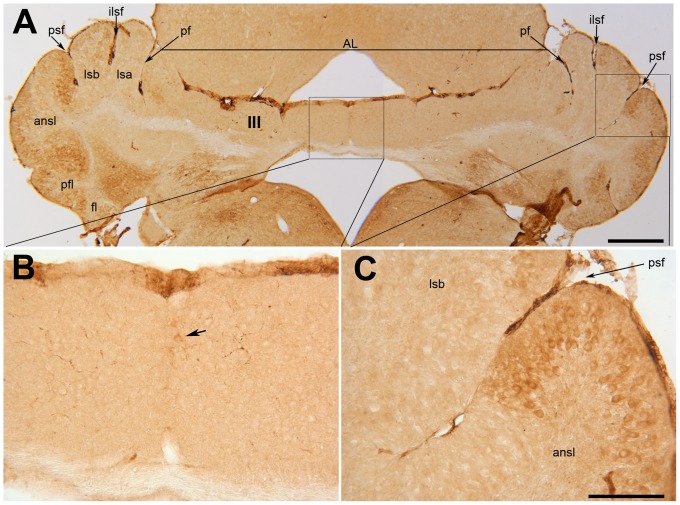
Transverse section through the anterior lobe of the *nax* cerebellum at P19 immunostained with ACP2. **A–C**) A section through lobule III of the *nax* cerebellum shows the underdeveloped cerebellum, particularly in the vermis, and the distinguishable primary fissure (pf) and lobules in the lateral cerebellum (A). Acp2 is clearly expressed in stripes in the hemispheres, seen at low magnification in A and at high magnification in C. Although there are no stripes in the putative vermis at low magnification (A), higher magnification shows a few Purkinje cells in the midline that express Acp2 (arrow) (B). Abbreviations: AL =  anterior lobe, ansl  = ansiform lobule, fl =  flocculus, ilsf  = intralobule simplex fissure, icf = intercrural fissure, Ls =  lobulus simplex, pfl =  paraflocculus, psf =  posterior superior fissure. Scale bars: A = 1 mm; C = 250 µm (B–C).

To further elucidate the patterning of the *nax* mutant cerebellum we used Acp2 as a stripe marker. Acp2 has recently been shown to be expressed in the adult mouse cerebellum in a pattern similar to ZII [23], therefore, it was used to explore the possibility of ZII+ stripes being absent in the anterior portion of the *nax* cerebellum. Acp2 was clearly expressed in stripes in the hemispheres of the *nax* cerebellum (Fig. 8A, C), but there were no obvious stripes in the putative vermis of the AZ. Despite the lack of stripes, it appeared as though a few Pcs in the midline were Acp2 immunopositive (Fig.8B).

**Figure 9:**
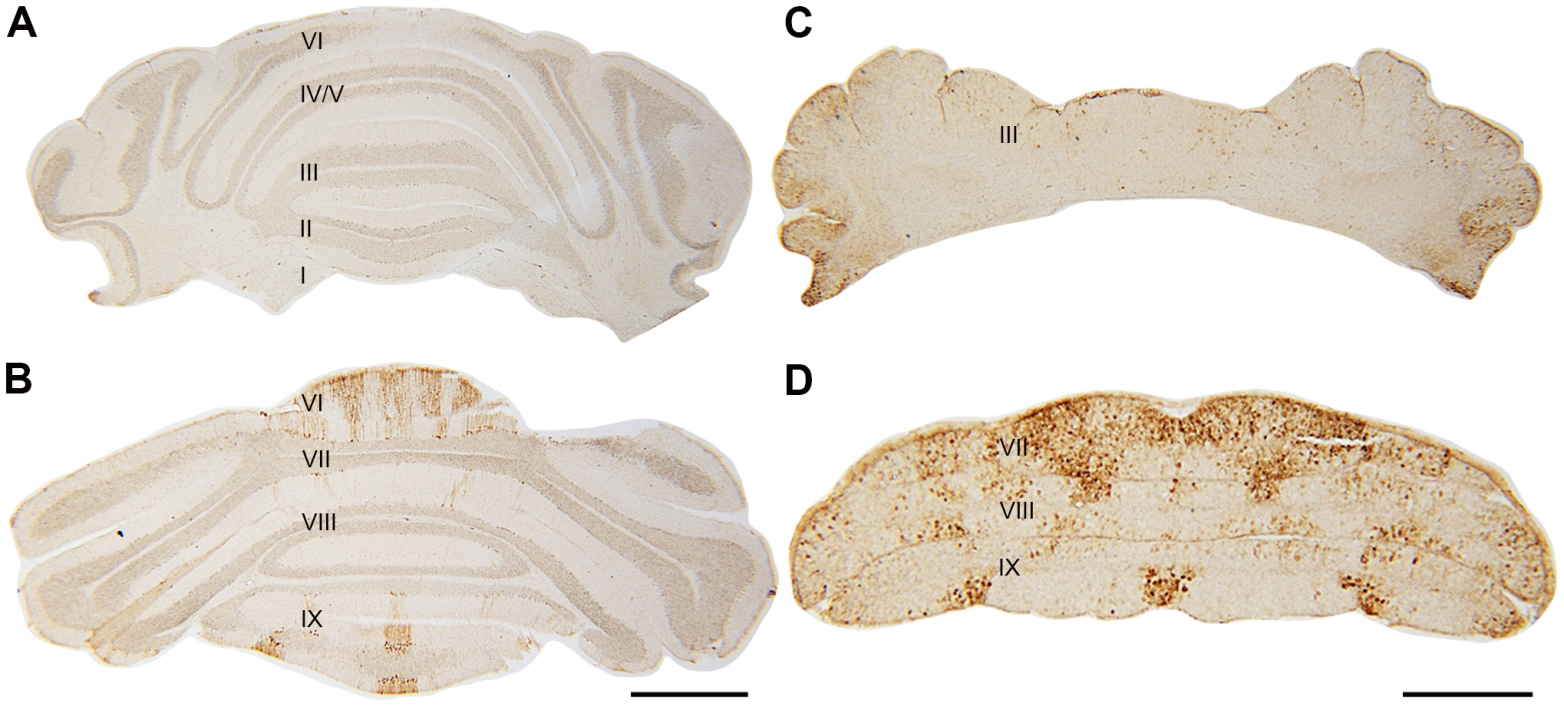
Transverse sections of wild type (A–B) and *nax* (C–D) cerebella at P19 immunostained with HSP25. Individual lobules are indicated in Roman numerals. **A–B**) HSP25 immunostaining is absent in the AZ and PZ, and expressed in parasagittal stripes in the CZ and NZ of the wild type cerebellum. **C–D**) The *nax* cerebellum shows similar staining to the wild type in the AZ and PZ, however, uniform expression occurs in the CZ (lobule VII). Lobule IX is elongated, but has a similar pattern of HSP25+ stripes as the wild type. Scale bar: B = 1 mm (A–B); D = 500 µm (C–D).

**Figure 10:**
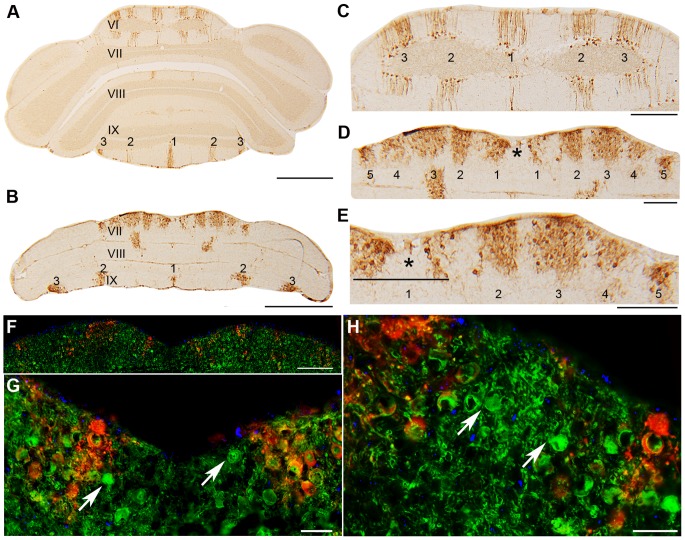
Transverse sections of wild type (A,C) and *nax* (B,D–E) cerebella at P22 immunostained with HSP25. **A,C**) HSP25 immunostaining shows an array of parasagittal stripes in the CZ and NZ of the wild type cerebellum, with high magnification of the CZ shown in **C**. **B, D– E**) The *nax* cerebellum shows similar staining to the wild type in the elongated lobule IX. In the CZ several parasagittal stripes are present, however, about five stripes occur on each side of the midline (asterisk; **D** is a higher magnification of **B**). At higher magnification **E**, the midline stripe (asterisk) is clearly absent, which could be due to the lack of complete fusion across the midline (asterisk) or from a split occurring later on during development. **F–H**) Transverse section of the nax cerebellum at P21 immunolabeled with HSP25 (red) and CaBP (green) (higher magnification in **G** and **H**). In the *nax* mutant, CaBP+ Purkinje cells (arrows) and DAPI+ nuclei (blue) are located between HSP25+ stripes in the CZ. Scale bars: A = 1 mm; B = 1 mm; C = 250 µm; D = 250 µm; E = 250 µm; F = 250 µm; G and H = 40 µm.

**Figure 11:**
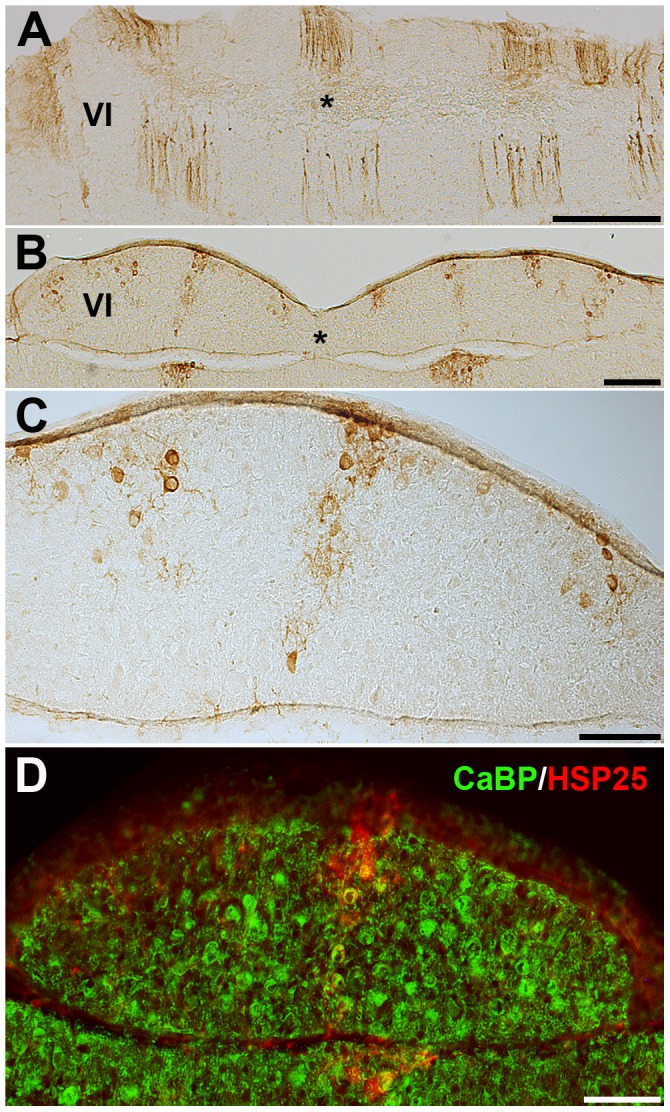
Transverse sections of wild type (A) and *nax* (B–E) cerebella at P26 immunostained with HSP25. **A**) HSP25 immunostaining shows an array of thick parasagittal stripes in the CZ of the wild type cerebellum (asterisks indicate midline). **B–C**) The P26 *nax* cerebellum has the same increased number of stripes in the CZ as the P22 cerebellum, however, the stripes appear narrower (asterisks indicate midline). Stripes are shown in the hemivermis at higher magnification in **C**. **D**) Transverse section of *nax* cerebellum at P26, stained with HSP25 and CaBP. CaBP immunopositive Purkinje cells are present on either side of HSP25+ stripes in the CZ. Scale bar: A = 200 µm; B = 200 µm; C, D = 100 µm.

To further characterize the organization of the vermis we used HSP25 as a marker of parasagittal stripes in the CZ and NZ of the adult mouse cerebellar vermis [31] (Fig. 9–11). At around P18–19, HSP25 expression was apparently uniform in the CZ or the HSP25- Pcs were weaker rather than completely negative, perhaps reflecting a delayed switch from global expression in the *nax* cerebellum (Fig. 9D). However, HSP25 was expressed in stripes in the NZ at this stage (Fig. 9D). Remarkably, at around P22–23, HSP25 expression in the CZ was heterogeneous, with an increased number of parasagittal stripes compared to the wild type (Fig. 10B, D–E). In the wild type, five stripes are usually present across the vermis of the CZ (Fig. 10A, C), however, in the *nax* mutant five distinct stripes were present in each half of the CZ vermis. Triple immunofluorescence staining at P21 showed that HSP25 negative Pc bodies were located between HSP25+ stripes in the CZ of the *nax* mutant (Fig.10 F–H, arrows). The NZ of the P22 *nax* mutant continued to express HSP25 in stripes comparable to the wild type. Normally the 3–5 HSP25+ stripes in the NZ occur only in the vermis, but in the unusually elongated lobule IX (ventral) of the *nax* mutant the stripes extended across the entire lobule. At around P26, HSP25 expression in the CZ still occurred in multiple stripes on either side of the midline in the *nax* mutant (Fig.11B asterisk), whereas stripes were symmetrical around a midline stripe in the wild type (Fig. 11A asterisk). The stripes in the P26 *nax* mutant appeared to be narrower and contained fewer Pcs (Fig. 11C) than those occurring at P22–23. Double immunofluorescence staining with HSP25 and CaBP revealed the continued presence of Pcs on either side of the HSP25+ stripes (Fig.11D).

## Discussion

In this study, we examined cerebellar anomalies in postnatal *nax* mutant mice and determined whether these defects were associated with alteration of the gene expression patterns normally found in the adult mouse cerebellum. The anteromedial cerebellum was severely disrupted compared to the posterolateral cerebellum, providing evidence that *Acp2* has a critical role in the process of cerebellar development, particularly in the rostral end where the greatest disruption in the zone and stripe phenotype occurred. This is consistent with previous results supporting the involvement of *Acp2* in cerebellar development due to early postnatal expression in the caudal mesencephalon and cerebellum [23].

Although all main lobules were present in the nax mutant, increased folding within lobules occurred in the anterior cerebellum. This abnormal anterior phenotype is similar to what has been seen in the rat cerebellum after methylazoxymethanol (MAM) treatment to ablate neonatal external granular cells [37]. The decreased presence of granule cells in the *nax* mutant suggests a shared process between these two similar phenotypes. For instance, it has been demonstrated that expression of the Shh target gene Gli1 is highest in the anteriomedial cerebellum and the level and length of this signaling influences foliation by regulating granule cell precursor proliferation [38], [39]. Interestingly, the increased folding in the *nax* mutant extended across the primary fissure into the rostral lobule VI vermis and lobule simplex a, indicating that common developmental mechanisms occur in these regions.

Examination of the external features of the *nax* cerebellum showed an underdeveloped or absent vermis and probably fusion of the two hemispheres at the midline. Histological analysis of transverse sections also supported that the vermis was partially absent or underdeveloped in the *nax* mutant and that this defect was most prominent anteriorly. Complete or partial absence of the vermis is a common defect in human diseases, such as tectocerebellar dysgraphia [40], Joubert anomaly (known as vermian aplasia) [41] and Gomez–Lopez-Hernandez syndrome (GLHS) [42], [43], and in several animal models, such as the cerebellar vermis defect (CVD) rat [44] and the *Lmx1a* mutant [17]. The fact that vermis defects are not unique to one mutant or condition suggests that there is a common error occurring in the process of vermis development or that the vermis is more vulnerable than the rest of the cerebellum during development.

To further elucidate the state of the vermis and gene expression patterns in the *nax* cerebellum, Pc compartmentation markers were used. Despite the reduced size, abnormal lobulation and Pc disruption, the zone and stripe pattern of the *nax* cerebellum appeared relatively normal, with two notable exceptions; the number of stripes present in the AZ was reduced and the HSP25 expression pattern in the CZ was remarkably abnormal.

### Reduced stripes in the AZ of *nax*

Both whole mount and section immunohistochemistry revealed that PLCB4-/ZII+ stripes appeared to be missing in the AZ of the *nax* cerebellum. However, this could be due to the fissures from increased lobule folding masking stripes or highlighting nonspecific reactions with pia mater. Additional staining with ACP2 (lobule III) showed almost no immunoreactivity medially, supporting the absence of PLCB4-/ZII+ stripes in the rostral AZ. ZII+ stripes became more obvious in sections through the caudal region of the AZ (lobule V), but there appeared to be fewer of these stripes compared to the wild type. This suggests that either some stripes (e.g. P2+, P3+ or P4+) on each side of the midline are missing, leaving a single ZII+ stripe, or due to a lack of PLCβ4+ Pcs all three ZII+ stripes are fused together to form a single wide stripe. To explain the absence of ZII+/PLCB4- stripes and potentially ZII-/PLCB4+ stripes in the AZ of the *nax* cerebellum we propose three possible hypotheses; A) failure of Pc subsets to disperse properly during development, B) alteration of the expression phenotype in subsets of Pcs during early development, or C) failure of Pc subsets to form during development.

Failure of particular subsets of Pcs to disperse properly has been reported to contribute to the appearance of cerebellar hypoplasia in other mouse mutants. Adult stripes are created through the perinatal rostrocaudal dispersion of embryonic Pc clusters, triggered by signaling through the Reelin pathway [15], [45]. Mice null for *Reelin* [19], [46], Reelin receptors [45], [47], or the adaptor protein disabled1 (*Dab1*) [46], [48], [49] show a deficiency in Pc dispersion, resulting in a large mass of ectopic Pcs deep in the white matter. Despite the apparent disorganization and ectopic location of Pcs, parasagittal stripes can still be outlined in these mutants. The *nax* cerebellum did not show the presence of ectopic Pcs deep in the cerebellar white matter; therefore, failure of Pc clusters to disperse entirely is an unlikely explanation for the potentially missing stripes in the AZ. Pcs were present in a disorganized multi-layer that invaded the molecular layer, which indicates that some interference with dispersion has occurred in the *nax* cerebellum. For example, transplantation of Pcs to host cerebella has shown that timing of dispersal affects the location in the cortex where Pcs migrate to, with those that are delayed in entering the cortex ending up in the molecular layer [50].

Specification of Pc phenotypes has been shown to occur early in development without influence from later interactions with climbing and mossy fibre afferents or other cerebellar cell types [51]–[54]. A recent study suggests that Pc birthdate is what specifies adult Pc phenotypes [55]. Thus, for the potentially missing stripes in the *nax* cerebellum to be a result of altered gene expression, Pcs could be born on a different day than they normally are. Another option is that *Acp2* affects phenotype specification early in development. For example, Ebf2 expression affects the ZII phenotype in mice, such that decreased Ebf2 causes ZII- Pcs to become ZII+, eliminating a portion of the striped pattern in the adult cerebellum [56], [57]. Perhaps Pcs in the *nax* mutant are born at the correct times, but the modified Acp2 protein directly or indirectly alters the gene expression profile in a subset of Pcs. In addition to having an important developmental role, Acp2 has been speculated to have neuroprotective properties [23]. However, in the *nax* mutant ZII+ and Acp2+ neurons appear to be the most affected cells. This suggests that the altered Acp2 protein present in *nax* could negatively affect Pc survival or proper phenotype specification early on.

### Abnormal expression pattern in the CZ of *nax*

To discover if the missing Pc stripes also extended into the CZ of the *nax* cerebellum we used the Pc compartmentation marker HSP25. At around P18–19, HSP25 expression in the *nax* cerebellum was uniform in the CZ and in stripes in the NZ, which is reminiscent of expression in the wild type at around P12 [58]. By around P22–23, expression in the CZ became striped (occurs by ∼P15–21 in wild type). This suggests that development in the *nax* cerebellum could be delayed, at least with respect to the HSP25 expression phenotype in the CZ. This is consistent with corticogenesis and development of the CZ normally occurring at a slower rate compared to the rest of the cerebellum (e.g. [13], [59]). The most interesting observation of HSP25 expression was the increase in the number of HSP25+ stripes in the CZ at around P22. Because specification of Pc phenotypes occurs very early on, the most likely explanation for this is inappropriate distribution of Pcs and/or misregulation in cluster to stripe formation during development. Another reason could be disruption in the down regulation of HSP25 by existing Pcs. Similar to the AZ, re-patterning of gene expression in the CZ is another possibility, as similar reorganization has been speculated in the Ebf2 null cerebellum [56].

Defects in lysosomal enzymes often contribute to lysosomal storage and neurodegeneration [5]. This raises the question of whether subsets of ZII+/Acp2+ Pcs within individual stripes could be dying, thus leading to the appearance of more stripes. Narrowing of the stripes from P22 to P26 is supportive of either Pc degeneration or continued down regulation of Hsp25 in the *nax* mutant. The density of Pcs was found to decrease significantly with age in the *nax* cerebellum suggesting the occurrence of Pc death. Immunohistochemistry with HSP25, CaBP and DAPI revealed the presence of cells between the HSP25+ Pcs, and despite abundant caspase-3 immunopositive cells in *nax* cerebellar cortex, none of them could be confirmed as Pcs. This suggests that classic apoptosis, as seen in the pcd mouse [60], may not be responsible for Pc death in the nax mutant. Further experiments would be needed to investigate Pc death in the *nax* cerebellum possibly through the autophagy or receptor interaction protein kinases 1 and 3 (RIP1 and RIP3)-dependent necroptosis pathways [61]. Therefore, the change of stripe number in the *nax* cerebellum could still be explained by Pc degeneration, however, altered patterns of gene expression during development cannot be excluded.

### PZ and NZ abnormalities

Gene expression in the PZ and NZ appeared less disrupted in comparison to the rest of the *nax* cerebellum. The most prominent defect in the caudal cerebellum was the mediolaterally elongated lobule IX. Immunohistochemistry using HSP25 revealed a similar number of stripes in lobule IX of *nax* compared to the wild type, however, these stripes extended across the entire lobule indicating an elongated vermis. This may indicate that lobule IX is fundamentally programmed to be comprised of the vermis and hemispheric component and in the *nax* mutant the hemispheric component is underdeveloped.

## Conclusion

In summary, we investigated the cerebellar anomalies associated with the *Acp2* mutation in mice. Malformation of the cerebellum was most severe rostromedially and milder caudolaterally suggesting that the role of *Acp2* is particularly critical in development of the rostral cerebellum. Extension of the disruption across the primary fissure into the rostral CZ also suggests the occurrence of common developmental processes in the AZ and CZ. The abnormal Pc organization in the *nax* cerebellar cortex could lead to the disrupted compartmentation organization, in which the stripes were more affected than the zones.

## References

[1] Lubke T, Lobel P, Sleat DE (2009) Proteomics of the lysosome. Biochim Biophys Acta 1793: 625–635. 18977398 10.1016/j.bbamcr.2008.09.018 PMC2684028

[2] Gottschalk S, Waheed A, Schmidt B, Laidler P, von Figura K (1989) Sequential processing of lysosomal acid phosphatase by a cytoplasmic thiol proteinase and a lysosomal aspartyl proteinase. EMBO J 8: 3215–3219. 2684640 10.1002/j.1460-2075.1989.tb08480.x PMC401441

[3] Vincent JB, Crowder MW, Averill BA (1992) Hydrolysis of phosphate monoesters: a biological problem with multiple chemical solutions. Trends Biochem Sci 17: 105–110. 1412693 10.1016/0968-0004(92)90246-6

[4] Makrypidi G, Damme M, Muller-Loennies S, Trusch M, Schmidt B, et al (2012) Mannose 6 dephosphorylation of lysosomal proteins mediated by acid phosphatases Acp2 and Acp5. Mol Cell Biol 32: 774–782. 22158965 10.1128/MCB.06195-11 PMC3272978

[5] Ballabio A, Gieselmann V (2009) Lysosomal disorders: from storage to cellular damage. Biochim Biophys Acta 1793: 684–696. 19111581 10.1016/j.bbamcr.2008.12.001

[6] Sato H, Keino H, Aono S, Semba R, Kashiwamata S (1987) Different behaviors among lysosomal enzymes in the cerebellum of jaundiced Gunn rats with cerebellar hypoplasia. J Neurochem 48: 1823–1825. 3033152 10.1111/j.1471-4159.1987.tb05742.x

[7] Ramaekers VT, Heimann G, Reul J, Thron A, Jaeken J (1997) Genetic disorders and cerebellar structural abnormalities in childhood. Brain 120 (Pt 10): 1739–1751. 9365367 10.1093/brain/120.10.1739

[8] Melquist S, Craig DW, Huentelman MJ, Crook R, Pearson JV, et al (2007) Identification of a novel risk locus for progressive supranuclear palsy by a pooled genomewide scan of 500,288 single-nucleotide polymorphisms. Am J Hum Genet 80: 769–778. 17357082 10.1086/513320 PMC1852701

[9] Pohl S, Mitchison HM, Kohlschutter A, van Diggelen O, Braulke T, et al (2007) Increased expression of lysosomal acid phosphatase in CLN3-defective cells and mouse brain tissue. J Neurochem 103: 2177–2188. 17868323 10.1111/j.1471-4159.2007.04920.x

[10] Saftig P, Hartmann D, Lullmann-Rauch R, Wolff J, Evers M, et al (1997) Mice deficient in lysosomal acid phosphatase develop lysosomal storage in the kidney and central nervous system. J Biol Chem 272: 18628–18635. 9228031 10.1074/jbc.272.30.18628

[11] Mannan AU, Roussa E, Kraus C, Rickmann M, Maenner J, et al (2004) Mutation in the gene encoding lysosomal acid phosphatase (Acp2) causes cerebellum and skin malformation in mouse. Neurogenetics 5: 229–238. 15503243 10.1007/s10048-004-0197-9

[12] Voogd J, Glickstein M (1998) The anatomy of the cerebellum. Trends Neurosci 21: 370–375. 9735944 10.1016/s0166-2236(98)01318-6

[13] Marzban H, Kim CT, Doorn D, Chung SH, Hawkes R (2008) A novel transverse expression domain in the mouse cerebellum revealed by a neurofilament-associated antigen. Neuroscience 153: 1190–1201. 18455884 10.1016/j.neuroscience.2008.02.036

[14] Ozol K, Hayden JM, Oberdick J, Hawkes R (1999) Transverse zones in the vermis of the mouse cerebellum. J Comp Neurol 412: 95–111. 10440712

[15] Apps R, Hawkes R (2009) Cerebellar cortical organization: a one-map hypothesis. Nat Rev Neurosci 10: 670–681. 19693030 10.1038/nrn2698

[16] Consalez GG, Hawkes R (2012) The compartmental restriction of cerebellar interneurons. Front Neural Circuits 6: 123. 23346049 10.3389/fncir.2012.00123 PMC3551280

[17] Sillitoe RV, George-Jones NA, Millen KJ, Hawkes R (2012) Purkinje cell compartmentalization in the cerebellum of the spontaneous mutant mouse dreher. Brain Struct Funct. 10.1007/s00429-012-0482-6 PMC4140632 23160833

[18] Armstrong C, Hawkes R (2001) Selective Purkinje cell ectopia in the cerebellum of the weaver mouse. J Comp Neurol 439: 151–161. 11596045 10.1002/cne.1339

[19] Edwards MA, Leclerc N, Crandall JE, Yamamoto M (1994) Purkinje cell compartments in the reeler mutant mouse as revealed by Zebrin II and 90-acetylated glycolipid antigen expression. Anat Embryol (Berl) 190: 417–428. 7887492 10.1007/BF00235488

[20] Beierbach E, Park C, Ackerman SL, Goldowitz D, Hawkes R (2001) Abnormal dispersion of a purkinje cell subset in the mouse mutant cerebellar deficient folia (cdf). J Comp Neurol 436: 42–51. 11413545

[21] Armstrong CL, Vogel MW, Hawkes R (2005) Development of Hsp25 expression compartments is not constrained by Purkinje cell defects in the Lurcher mouse mutant. J Comp Neurol 491: 69–78. 16127699 10.1002/cne.20703

[22] Geier C, Kreysing J, Boettcher H, Pohlmann R, von Figura K (1992) Localization of lysosomal acid phosphatase mRNA in mouse tissues. J Histochem Cytochem 40: 1275–1282. 1506664 10.1177/40.9.1506664

[23] Bailey K, Rahimi Balaei M, Mehdizadeh M, Marzban H (2013) Spatial and Temporal Expression of Lysosomal Acid Phosphatase 2 (ACP2) Reveals Dynamic Patterning of the Mouse Cerebellar Cortex. Cerebellum 12: 870–881. 23780826 10.1007/s12311-013-0502-y

[24] Tano D, Napieralski JA, Eisenman LM, Messer A, Plummer J, et al (1992) Novel developmental boundary in the cerebellum revealed by zebrin expression in the lurcher (Lc/+) mutant mouse. J Comp Neurol 323: 128–136. 1430312 10.1002/cne.903230111

[25] Baimbridge KG, Miller JJ (1982) Immunohistochemical localization of calcium-binding protein in the cerebellum, hippocampal formation and olfactory bulb of the rat. Brain Res 245: 223–229. 6751467 10.1016/0006-8993(82)90804-6

[26] De Camilli P, Miller PE, Levitt P, Walter U, Greengard P (1984) Anatomy of cerebellar Purkinje cells in the rat determined by a specific immunohistochemical marker. Neuroscience 11: 761–817. 6330609 10.1016/0306-4522(84)90193-3

[27] Hawkes R, Herrup K (1995) Aldolase C/zebrin II and the regionalization of the cerebellum. J Mol Neurosci 6: 147–158. 8672398 10.1007/BF02736761

[28] Brochu G, Maler L, Hawkes R (1990) Zebrin II: a polypeptide antigen expressed selectively by Purkinje cells reveals compartments in rat and fish cerebellum. J Comp Neurol 291: 538–552. 2329190 10.1002/cne.902910405

[29] Sarna JR, Marzban H, Watanabe M, Hawkes R (2006) Complementary stripes of phospholipase Cbeta3 and Cbeta4 expression by Purkinje cell subsets in the mouse cerebellum. J Comp Neurol 496: 303–313. 16566000 10.1002/cne.20912

[30] Marzban H, Chung S, Watanabe M, Hawkes R (2007) Phospholipase Cbeta4 expression reveals the continuity of cerebellar topography through development. J Comp Neurol 502: 857–871. 17436294 10.1002/cne.21352

[31] Armstrong CL, Krueger-Naug AM, Currie RW, Hawkes R (2000) Constitutive expression of the 25-kDa heat shock protein Hsp25 reveals novel parasagittal bands of purkinje cells in the adult mouse cerebellar cortex. J Comp Neurol 416: 383–397. 10602096 10.1002/(sici)1096-9861(20000117)416:3<383::aid-cne9>3.0.co;2-m

[32] Marzban H, Hawkes R (2007) Fibroblast growth factor promotes the development of deep cerebellar nuclear neurons in dissociated mouse cerebellar cultures. Brain Res 1141: 25–36. 17300764 10.1016/j.brainres.2007.01.031

[33] Fortin M, Marchand R, Parent A (1998) Calcium-binding proteins in primate cerebellum. Neurosci Res 30: 155–168. 9579649 10.1016/s0168-0102(97)00124-7

[34] Bastianelli E (2003) Distribution of calcium-binding proteins in the cerebellum. Cerebellum 2: 242–262. 14964684 10.1080/14734220310022289

[35] Kemp K, Gray E, Wilkins A, Scolding N (2012) Purkinje cell fusion and binucleate heterokaryon formation in multiple sclerosis cerebellum. Brain 135: 2962–2972. 22975392 10.1093/brain/aws226

[36] Sillitoe RV, Hawkes R (2002) Whole-mount immunohistochemistry: a high-throughput screen for patterning defects in the mouse cerebellum. J Histochem Cytochem 50: 235–244. 11799142 10.1177/002215540205000211

[37] Ji Z, Hawkes R (1996) Partial ablation of the neonatal external granular layer disrupts mossy fiber topography in the adult rat cerebellum. J Comp Neurol 371: 578–588. 8841911 10.1002/(SICI)1096-9861(19960805)371:4<578::AID-CNE7>3.0.CO;2-1

[38] Corrales JD, Rocco GL, Blaess S, Guo Q, Joyner AL (2004) Spatial pattern of sonic hedgehog signaling through Gli genes during cerebellum development. Development 131: 5581–5590. 15496441 10.1242/dev.01438

[39] Corrales JD, Blaess S, Mahoney EM, Joyner AL (2006) The level of sonic hedgehog signaling regulates the complexity of cerebellar foliation. Development 133: 1811–1821. 16571625 10.1242/dev.02351

[40] Krishnamurthy S, Kapoor S, Sharma V, Prakash A (2008) Tectocerebellar dysraphia and occipital encephalocele: an unusual association with abdominal situs inversus and congenital heart disease. Indian J Pediatr 75: 1178–1180. 18810345 10.1007/s12098-008-0183-6

[41] Joubert M, Eisenring JJ, Robb JP, Andermann F (1969) Familial agenesis of the cerebellar vermis. A syndrome of episodic hyperpnea, abnormal eye movements, ataxia, and retardation. Neurology 19: 813–825. 5816874 10.1212/wnl.19.9.813

[42] Whetsell W, Saigal G, Godinho S (2006) Gomez-Lopez-Hernandez syndrome. Pediatr Radiol 36: 552–554. 16607507 10.1007/s00247-006-0150-1

[43] Fernandez-Jaen A, Fernandez-Mayoralas DM, Calleja-Perez B, Munoz-Jareno N, Moreno N (2009) Gomez-Lopez-Hernandez syndrome: two new cases and review of the literature. Pediatr Neurol 40: 58–62. 19068257 10.1016/j.pediatrneurol.2008.10.001

[44] Kuwamura M, Ishida A, Yamate J, Kato K, Kotani T, et al (1997) Chronological and immunohistochemical observations of cerebellar dysplasia and vermis defect in the hereditary cerebellar vermis defect (CVD) rat. Acta Neuropathol 94: 549–556. 9444356 10.1007/s004010050749

[45] Larouche M, Beffert U, Herz J, Hawkes R (2008) The Reelin receptors Apoer2 and Vldlr coordinate the patterning of Purkinje cell topography in the developing mouse cerebellum. PLoS One 3: e1653. 18301736 10.1371/journal.pone.0001653 PMC2242849

[46] Goldowitz D, Cushing RC, Laywell E, D'Arcangelo G, Sheldon M, et al (1997) Cerebellar disorganization characteristic of reeler in scrambler mutant mice despite presence of reelin. J Neurosci 17: 8767–8777. 9348346 10.1523/JNEUROSCI.17-22-08767.1997 PMC6573071

[47] Hack I, Hellwig S, Junghans D, Brunne B, Bock HH, et al (2007) Divergent roles of ApoER2 and Vldlr in the migration of cortical neurons. Development 134: 3883–3891. 17913789 10.1242/dev.005447

[48] Hiesberger T, Trommsdorff M, Howell BW, Goffinet A, Mumby MC, et al (1999) Direct binding of Reelin to VLDL receptor and ApoE receptor 2 induces tyrosine phosphorylation of disabled-1 and modulates tau phosphorylation. Neuron 24: 481–489. 10571241 10.1016/s0896-6273(00)80861-2

[49] Feng L, Cooper JA (2009) Dual functions of Dab1 during brain development. Mol Cell Biol 29: 324–332. 18981215 10.1128/MCB.00663-08 PMC2612505

[50] Carletti B, Williams IM, Leto K, Nakajima K, Magrassi L, et al (2008) Time constraints and positional cues in the developing cerebellum regulate Purkinje cell placement in the cortical architecture. Dev Biol 317: 147–160. 18384765 10.1016/j.ydbio.2008.02.005

[51] Seil FJ, Johnson ML, Hawkes R (1995) Molecular compartmentation expressed in cerebellar cultures in the absence of neuronal activity and neuron-glia interactions. J Comp Neurol 356: 398–407. 7642801 10.1002/cne.903560307

[52] Leclerc N, Gravel C, Hawkes R (1988) Development of parasagittal zonation in the rat cerebellar cortex: MabQ113 antigenic bands are created postnatally by the suppression of antigen expression in a subset of Purkinje cells. J Comp Neurol 273: 399–420. 2463281 10.1002/cne.902730310

[53] Wassef M, Sotelo C, Thomasset M, Granholm AC, Leclerc N, et al (1990) Expression of compartmentation antigen zebrin I in cerebellar transplants. J Comp Neurol 294: 223–234. 2332530 10.1002/cne.902940207

[54] Sillitoe RV, Vogel MW, Joyner AL (2010) Engrailed homeobox genes regulate establishment of the cerebellar afferent circuit map. J Neurosci 30: 10015–10024. 20668186 10.1523/JNEUROSCI.0653-10.2010 PMC2921890

[55] Namba K, Sugihara I, Hashimoto M (2011) Close correlation between the birth date of Purkinje cells and the longitudinal compartmentalization of the mouse adult cerebellum. J Comp Neurol 519: 2594–2614. 21456012 10.1002/cne.22640

[56] Chung SH, Marzban H, Croci L, Consalez GG, Hawkes R (2008) Purkinje cell subtype specification in the cerebellar cortex: early B-cell factor 2 acts to repress the zebrin II-positive Purkinje cell phenotype. Neuroscience 153: 721–732. 18403128 10.1016/j.neuroscience.2008.01.090

[57] Croci L, Chung SH, Masserdotti G, Gianola S, Bizzoca A, et al (2006) A key role for the HLH transcription factor EBF2COE2,O/E-3 in Purkinje neuron migration and cerebellar cortical topography. Development 133: 2719–2729. 16774995 10.1242/dev.02437

[58] Armstrong CL, Krueger-Naug AM, Currie RW, Hawkes R (2001) Expression of heat-shock protein Hsp25 in mouse Purkinje cells during development reveals novel features of cerebellar compartmentation. J Comp Neurol 429: 7–21. 11086286 10.1002/1096-9861(20000101)429:1<7::aid-cne2>3.0.co;2-q

[59] Vastagh C, Vig J, Hamori J, Takacs J (2005) Delayed postnatal settlement of cerebellar Purkinje cells in vermal lobules VI and VII of the mouse. Anat Embryol (Berl) 209: 471–484. 15887047 10.1007/s00429-005-0458-x

[60] Chakrabarti L, Eng J, Ivanov N, Garden GA, La Spada AR (2009) Autophagy activation and enhanced mitophagy characterize the Purkinje cells of pcd mice prior to neuronal death. Mol Brain 2: 24. 19640278 10.1186/1756-6606-2-24 PMC2729476

[61] Fayaz SM, Suvanish Kumar VS, Rajanikant GK (2014) Necroptosis: Who Knew There were so Many Interesting Ways to Die? CNS Neurol Disord Drug Targets 13: 42–51. 24152329 10.2174/18715273113126660189

